# Analysis of Mechanical Properties of Welded Joint Metal from TPP Steam Piping after Its Operational Degradation and Hydrogenation

**DOI:** 10.3390/ma16247520

**Published:** 2023-12-05

**Authors:** Volodymyr Hutsaylyuk, Oleksandra Student, Pavlo Maruschak, Halyna Krechkovska, Olha Zvirko, Lesya Svirska, Ivan Tsybailo

**Affiliations:** 1 Faculty of Mechanical Engineering, Military University of Technology, Gen. S. Kaliskiego Str. 2, 00-908 Warsaw, Poland; volodymyr.hutsaylyuk@wat.edu.pl; 2 Department of Diagnostics of Materials Corrosion-Hydrogen Degradation, Karpenko Physico-Mechanical Institute of the NAS of Ukraine, Naukova Str. 5, 79060 Lviv, Ukraine; oleksandrastudent1@gmail.com (O.S.); krechkovskahalyna@gmail.com (H.K.); olha.zvirko@gmail.com (O.Z.); lesyasvirska@gmail.com (L.S.); tsybailo.14@gmail.com (I.T.); 3 Department of Industrial Automation, Ternopil National Ivan Puluj Technical University, Ruska Str. 56, 46001 Ternopil, Ukraine; 4 Department of Materials Science and Engineering, Lviv Polytechnic National University, Ustyianovycha Str. 5, Building 10, Room 28a, 79013 Lviv, Ukraine

**Keywords:** heat-resistant steel, welded joint, thermal power plant, hydrogenation, operational degradation, mechanical properties

## Abstract

In this paper, the mechanical properties of various zones of the welded joints of a heat-resistant steel 15Kh1M1F in different states (in the initial state, after an operation on the main steam piping of a thermal power plant (TPP) for 23 years) were determined, and the fracture surfaces were analyzed using scanning electron microscopy (SEM) images. The effect of hydrogen electrolytic charging on mechanical behavior and fracture mechanism was also studied. The long-term operation of welds resulted in a higher degradation degree of the weld metal compared to the base one, indicated by the deterioration of mechanical properties: decrease in hardness, strength characteristics, and reduction in area, which was accompanied by an atypical increase in elongation at fracture. All studied zones of the operated welded joints were characterized by higher hydrogen content, 2.5–3 times higher than that in the initial state. Additional hydrogen charging of the weld joint metal led to a decrease in the strength and ductility characteristics, more significantly for the operated weld compared with the non-operated one. This justified the possibility of using short-term tests of hydrogenated WM in the air to assess the degree of its damage during operation on a steam piping.

## 1. Introduction

Degradation of metal weld joints after long-term operation is one of the main reasons for reducing the reliability and operational safety of thermal power plants (TPPs) equipment [1]. However, such equipment should have as long a service life as possible. Its duration can be extended after assessing the structural and mechanical condition of its components [2]. The premature failure of welded joints is a serious problem in thermal power engineering because the creep resistance of weld metals decreases faster than that of base metals due to high temperatures and applied stresses [3,4,5]. Areas of welds are potentially dangerous because they are more structurally heterogeneous than the surrounding metal and are zones of localization of stresses and deformations [6,7,8].

The main factors of the degradation of heat-resistant steels operated at TPPs include high temperature and stress, supplemented by the influence of a hydrogen-inducing technological environment [9,10,11,12,13,14,15,16,17,18,19,20,21,22,23]. During the long-term operation of high- and low-alloy heat-resistant steels, the carbides form and coagulate along the grain boundaries. It was shown that the nature of carbide phases [24] and the shape and size of non-metallic inclusions [25,26] significantly impact steels’ ability to hydrogen and, consequently, hydrogen embrittlement. In particular, in the structure of heat-resistant steel for reactor vessels alloyed with vanadium, nano-sized carbides (Mo, V) C are released, which are traps for hydrogen absorbed by the metal and thereby reduce its activity in initiating cracks inside the metal [27]. Visualization of hydrogen diffusion paths in the steel structure made it possible to show an increased discharge of hydrogen around inclusions [28]. Easy capture of hydrogen (absorbed by steel during long-term operation) should promote the decohesion of both non-metallic inclusions and carbides from the surrounding matrix. As a result, the number of internal defects in the steel structure should increase. Their sizes will be comparable to the sizes of particles whose decohesion from the matrix has already occurred. Metal deformation further weakens the cohesion of carbides with the surrounding ferrite matrix [19]. The discrepancy between the elastic characteristics of carbides and the matrix phase directly affects the deformation in local zones near their interfaces [29]. This promotes the formation of intergranular pores and weakens the cohesion of neighboring grains. As a result, the fracture of the structural elements is facilitated due to creep (especially in a hydrogenating medium [30,31,32]) and thermal cycling (in particular, due to frequent shutdowns of the technological process) [14,33,34].

In composition and properties, welded joints (as an integral part of complex structures) are usually close to the base metal [35,36]. In critical structures, their properties are even slightly better than those of the base metal. Therefore, the established features of high-temperature degradation of heat-resistant steels (as the base metal [37]) should also apply to the weld metal. Hydrogenation is also considered a technological impact factor that contributes to the degradation of steels in structural elements during long-term operation. In general, hydrogenation of heat-resistant steels (like most structural materials) contributes to their embrittlement [38,39,40]. This is especially important during frequent shutdowns of technological processes [14] when the solubility of hydrogen in the metal decreases and its forced desorption occurs. Based on the relationship between the hydrogen content in 2.25Cr1Mo0.25V steel and its characteristics during tensile tests, the loss of its ductility under the influence of absorbed hydrogen was used to assess its susceptibility to hydrogen embrittlement [38]. Tests of vanadium-modified steel 2.25Kh1Mo0.25V and its welded joint during high-temperature tensile and creep tests in the temperature range 350–500 °C showed that, due to smaller grains, WM has higher strength and lower ductility than BM. At the same time, short-term creep tests at a temperature of 550 °C showed that WM has a higher creep resistance than BM [35]. However, it is known that even during short-term high-temperature (more than 400 °C) tensile tests of the weld metal, its yield strength decreases faster than that of the base metal [41]. There is no reason to believe that during long-term high-temperature operation of welded structures, this trend of accelerated degradation of the weld metal (relative to the base metal) will be reversed. The results of the analyzed experimental studies were mainly related to materials in an unused state. However, it is very important to know the current properties of materials at any stage of their operation. Our studies of creep in gaseous hydrogen showed an increase in the creep rate of the exploited steel compared to steel in the initial state [30]. It is difficult to hope that the properties of the weld metal will improve after long-term use.

Currently, there are no standards for a comprehensive assessment of the actual state of operational weld joints that would take into account changes in the metal state at the micro level, as well as changes in its mechanical properties at the macro level [42]. This makes it difficult to predict the kinetics of damage formation in welded joints and classify structural and mechanical changes in the metal, which is necessary to predict the residual life of the technological equipment of TPPs. Creep tests are quite expensive and require large cuttings of metal from structural elements. Therefore, it is important to find ways to increase the sensitivity of traditional properties at short-term tensile tests to the deterioration of the metal state of exploited welds. This made it possible to estimate at least the relative change in their properties as a result of operation. Consequently, assessing the current state of the weld metal after its long-term operation in real high-temperature technological processes is a very urgent task for many industries.

The purpose of this work is to experimentally substantiate the possibility of assessing the degree of degradation of the weld metal of heat-resistant steel after its long-term operation on the main steam piping of a TPP based on traditional tensile tests of smooth samples in air, but after their preliminary electrolytic hydrogenation with subsequent determination of the ratio of the corresponding strength characteristics in the operating and the original weld metal, characterizing the degree of its degradation.

## 2. Materials and Methods

Metals of two circumferential welded joints (WJ) on pipes with a diameter of ~325 mm and a wall thickness of ~60 mm were examined in the initial state (as a model WJ) and after ~2 × 10^5^ h of high-temperature (540 °C) operation on the vertical section of the TPP main steam piping. In welded joints, 15Kh1M1F steel of two variants was used as the base metal (BM): BM1 (wt.%: 0.14 C; 1.33 Cr; 0.91 Mo; 0.22 V; 0.18 Ni; 0.32 Si; 0.70 Mn; 0.023 S; 0.024 P) and BM2 (wt.%: 0.16 C; 1.39 Cr; 0.97 Mo; 0.29 V; 0.2 Ni; 0.30 Si; 0.67 Mn; 0.023 S; 0.025 P). The model WJ was made by manual multi-pass electric arc welding with TML-3U electrodes using the technology for welding thick-walled pipes, which is effective during the installation of the steam piping system. Thus, in both cases, the pipes were welded in reverse polarity mode with direct current. The first three passes were performed with electrodes with a diameter of 3 mm and the subsequent ones with a diameter of 4 mm. The welding current at these stages was 105 and 150 A, arc voltage 28 and 30 V, heat input 4.6 and 5.3 kJ/mm, respectively. To obtain a model weld, meter-long sections of pipe with machined edges were used to form a V-shaped groove. Before welding, the pipes were heated to a temperature of 300 °C. In each pass, rollers with a thickness of 4–6 mm were applied along the perimeter of the WJ. Upon completion of welding, the WJ was cooled under heat-insulating mats for 6 h. After this, the WJ was thermally treated with repeated heating to 600 °C at a rate not exceeding 200 °C/h and with a subsequent increase in temperature to 735 ± 15 °C with a lower heating rate (100 °C/h) and exposure for 3 h. The BM and weld metal (WM) chemical compositions were determined on a SPECTROMAX LMF 0.5 spark atomic emission spectrometer using 20 × 20 × 5 mm specimens with plane-parallel surfaces. According to the chemical composition, the WM of both WJs corresponded to steel 09Kh1MF (Table 1). In the initial state, the strength of the WM exceeded, and the ductility was compared with the corresponding characteristics of the BM, which satisfied the requirements for the properties of WJ (Table 2).

The technical state of the analyzed variants of WJ was assessed by analysis of the following characteristics. First of all, this is the Brinell hardness (HB), measured at a depth of 5 mm from the outer surface of the pipes on polished plane-parallel specimens cut across the WJ. Hardness values were averaged based on approx. 10 measurements. Data scatter was within 5%. The mechanical characteristics were determined on smooth cylindrical specimens of 5 mm in diameter, cut from BM and WM, using a UME-10T tensile testing machine (LLC Tochmashprobor, Armavir, Russia). To evaluate the impact strength of the materials under study, Charpy specimens (10 × 10 × 55 mm) with a sharp V-shaped notch (the notch radius was 0.25 mm) were tested using an IO-5003 type pendulum impact machine (LLC ZIP, Ivanovo, Russia). All specimens from both WJs that were used to assess the mechanical properties of the metals were cut out from their different zones. To produce them, pipes with circumferential welds were cut into diametrically oriented rings up to 12 mm thick, from which radially oriented samples were then made. To determine the location of these rings relative to the weld zones, its macro-etching on the surface of the welded pipes was used. The cross-section of the ring in the WM zone was centered relative to the axis of the weld, so the specimens made further from it were also oriented along this axis. The notches on impact specimens were cut in a circumferential direction to a depth of 2 mm. In this case, the direction of specimen failure coincided with the direction of the circumferential weld. All values of the mechanical characteristics were averaged based on the results of three to five tests; the relative error did not exceed 7%.

Mechanical characteristics under tension were determined for WJ in the initial state (specimens were cut from the model WJ), WJ after ~2 × 10^5^ h of operation on the steam piping, and both of these variants after additional electrolytic charging with hydrogen. The tensile tests were performed in air at a strain rate of 3 × 10^−3^ s^−1^.

For hydrogen charging, the specimens were kept for 15 min in sulfuric acid (pH0) with the addition of thiourea (2 g/L) at a cathodic polarization current density of 50 mA/cm^2^. To remove electrolyte residues, the specimens were washed and dried and then tensile tested in air 5 min after completion of electrolytic hydrogenation. Thus, the role of internal hydrogen, which could be absorbed by the metal during operation on the steam piping, was evaluated. 

The integral content of diffusible (at a temperature up to ~900 °C) hydrogen in BM and WM was determined by chromatographic method in compliance with the requirements of the regulatory document [43].

Digital fractographic images were recorded in the central part of the fractures of the WM specimens (where the fracture is usually initiated during tensile tests of smooth samples in the air) in the initial state and after operation using a Hitachi S-2600N (Hitachi Scientific Instruments, Tokyo, Japan) SEM. To analyze and compare the fracture features caused by operational degradation of the WM and its hydrogenation, from 9 to 12, typical fractographic images of both WM variants were recorded. 

## 3. Results and Discussion

### 3.1. Changes in the Mechanical Properties of the Studied Materials Due to Operational Degradation 

To assess the state of the WJ metal at different stages of its operation, the regulatory documents of Ukraine’s thermal power industry mainly recommend mechanical properties such as hardness, strength, ductility, and impact strength [44]. To compare the sensitivity of the recommended mechanical properties to high-temperature degradation, the data for WM in the initial state and after its long-term operation were analyzed. 

#### 3.1.1. Sensitivity of Hardness to Weld Joint Degradation 

The hardness of WM in the initial state (the specimen was cut from the model WJ) exceeded the hardness of BM (steel 15Kh1M1F) almost throughout the entire thickness of the pipe wall, which is in accordance with data in [45]. In the deeper layers of the pipe wall, the hardness of WM in the initial state gradually decreased to 180 … 210 HB. However, at all levels of measurement, the hardness of the WM remained consistently above the level of 150–170 HB characteristic of the BM (BM1 and BM2 on both sides of the WM) and not lower than the value of 150 HB, which is regulated for steam piping. According to the measurements, at a depth of 5 mm from the pipe’s outer surface, the WM’s hardness varied within 230–250 HB. Unfortunately, there is no data on variations of hardness through the pipe wall at the beginning of its operation. At the same time, the hardness of WM on the outer surface of the pipe before its operation exceeded the hardness of BM in the initial state and corresponded to the hardness of the model WJ obtained in compliance with the welding technology used at that time. Therefore, it was assumed that the WM of the model WJ corresponded to the WM of the operated WJ at the beginning of its operation.

**Figure 1:**
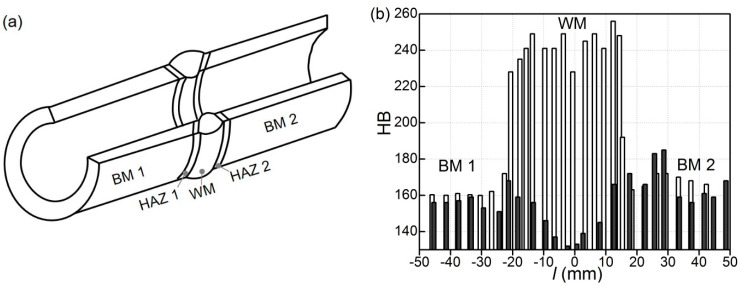
The scheme of the location of different zones of the circumferential WJ (**a**) and hardness HB (**b**) of the metal of these zones in the initial state (white bars) and after ~2 × 10^5^ h of operation on the TPP main steam piping (black), measured across the WJ at different distances *l* from their axis at a depth of 5 mm from the outer surface of pipes.

Long-term operation of the WJ on the main steam piping of the TPP practically did not affect the hardness of the BM (Figure 1b). This indicates a low sensitivity of hardness to BM degradation, and it is consistent with the low sensitivity of the strength characteristics of BM to operational degradation [46]. At the same time, the HB hardness of the operated WM more strongly decreased and became even lower than the hardness of the BM. This trend persisted over almost the entire thickness of the pipe wall. The BM hardness still exceeded the WM hardness only near the inner surface of the operating pipe. The maximum effect of WM softening due to operational degradation was found near the outer surface of the pipe, where maximum tensile thermal stresses acted during block shutdowns, according to [34]. These stresses additionally contributed to the formation of damage in the WM due to creep. Thus, at a depth of 5 mm from the outer surface of the pipe, the hardness of the WM decreased from ~245 HB in the initial state to ~140 HB after its operation (Figure 1b). As a result, the hardness of WM decreased by ~44% due to the in-service degradation. However, more importantly, the hardness of the WM became lower than the hardness of the BM and the lower limit value for steam piping (~150 HB). Therefore, despite the low sensitivity of hardness to the operational degradation of BM, its sensitivity to the degradation of WM was quite high. 

#### 3.1.2. Changes in Strength, Ductility, and Toughness Characteristics Due to Weld Metal Degradation 

**Figure 2:**
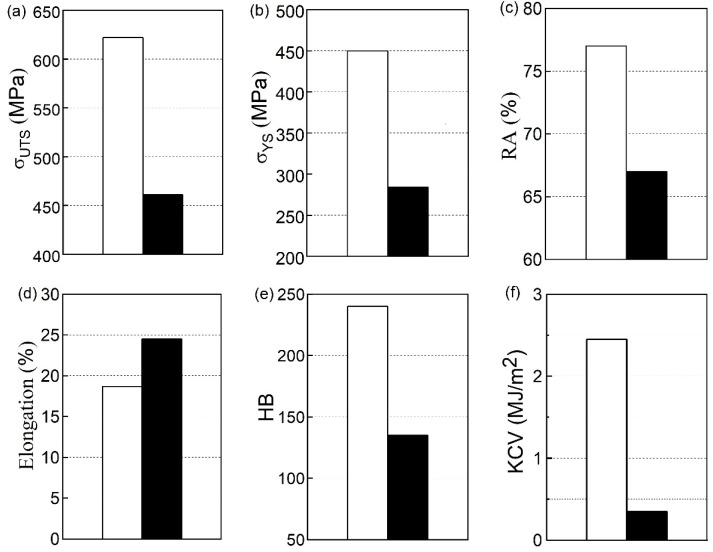
Strength (σ_UTS_—(**a**), σ_YS_—(**b**)) and ductility (RA—(**c**), elongation—(**d**)), hardness HB (**e**) and impact strength KCV (**f**) of the WM in the initial state (white bars) and after ~2 × 10^5^ h of operation on the TPP main steam piping (black).

Almost all the most commonly used mechanical properties of WM (except elongation) decreased to some extent after its operation on a steam piping (Figure 2). Moreover, they were quite sensitive to changes in the state of the WM as a result of long-term operations. This is despite the previously noted low sensitivity of hardness, strength, and plasticity in tension to the degradation of BM [45]. A noticeable decrease in the tensile strength (26%) of the WM during operation indicates a decrease in its cohesive strength and tear resistance (Figure 2a). The decrease in its yield strength reached 44% and was the same as the decrease in HB hardness (44%). This is quite an expected result since both indicators characterize the WM’s ability to deform (Figure 2b,e). Their decrease also indicates a decrease in the ability of the steam piping element with a WJ to resist operational stresses. Therefore, it can be dangerous for its further operation on the steam piping.

Regarding the ductile characteristics of WM, an atypical opposite trend of their change due to operational degradation was revealed. Usually, the ductility characteristics of the metal in the initial state change in a similar way (both indicators either increase or decrease). This trend of their change is justified by the influence of traditional factors (alloying, heat treatment, deformation, etc.). Moreover, their change usually correlates with changes in the characteristics of resistance to brittle fracture. However, after long-term operation, both ductile characteristics of the WM changed in opposite directions. Despite a slight decrease in the reduction by area of the operated WM (by 9%), its elongation unexpectedly increased by ~33% (Figure 2c,d). The recorded increase in the elongation of the degraded WM was explained by operational damage randomly scattered in the bulk of the WM along the entire working length of the specimen. These damages were extended in the direction of the load applied to the specimen during its tensile test. It was assumed that these damages additionally contributed to an increase in the elongation of the specimen already at the stage of its uniform plastic deformation and, as a consequence, its total elongation [45]. Therefore, it is impossible to characterize the plasticity of a long-term operated WM using both indicators of plasticity simultaneously.

As noted above, the tensile mechanical properties of the used WM changed significantly, but its impact strength KCV (as a characteristic of brittle fracture resistance) decreased even more (Figure 2f). The decrease in their KCV values relative to the corresponding values for WM in the initial state reached 86%. Therefore, an almost twofold increase in the sensitivity to WM degradation of the KCV indicator was recorded compared to traditional mechanical characteristics (hardness, strength, and ductility). This indicates an increasing propensity of WM in operation to brittle fracture. Low resistance to brittle fracture is usually characteristic of materials with high hardness and strength. Therefore, the identified anomalous trends in the change in the mechanical properties of WM were considered as features of its degradation during operation. Indeed, as a rule, the trend of change in strength characteristics and impact strength is opposite to that observed for the exploited WM. This trend remains valid, and metal with lower strength is characterized by higher impact strength after exposure to the metal heat treatment, alloying, and deformation. The factor of the high-temperature degradation of WM is an exception to this rule.

The opposite change in plasticity characteristics, on the one hand, and the simultaneous decrease in strength characteristics and resistance to brittle fracture, on the other hand, indicates atypical changes in various mechanical properties due to high-temperature degradation of WM. Indeed, an increase in the elongation of the exploited WM is consistent with a decrease in its strength, and a decrease in the RA value is consistent with a decrease in the resistance to brittle fracture. Typically, elongation and RA characteristics exhibit different sensitivity to hydrogen embrittlement of the metal. Usually, preference is given to reducing the specimen’s area since this indicator changes more significantly under the action of hydrogen. The same trend towards a decrease in the RA and KCV values of the degraded WM was considered a sign of its increased tendency to hydrogen embrittlement, which promotes brittle fracture.

#### 3.1.3. Comparison of Mechanical Characteristics Based on Their Sensitivity to Operational Degradation of the Weld Metal

**Figure 3:**
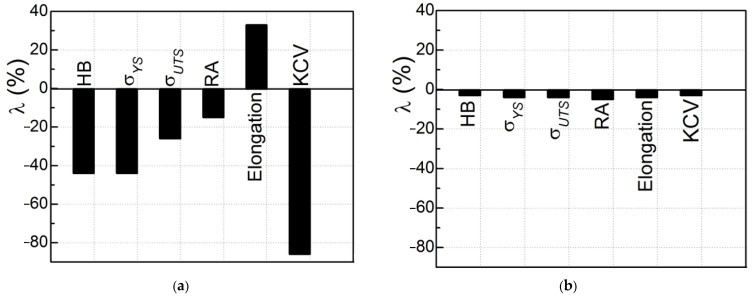
Sensitivity of various mechanical properties (hardness HB, tensile strength σ_UTS_ and yield strength σ_YS_, reduction in area RA, elongation and impact strength (KCV)) to high-temperature degradation of WM (**a**) and BM 2 (**b**). The ratio λ characterizes the change in each characteristic for both exploited metals relative to their corresponding characteristics in the initial state.

Based on a generalization of the obtained results, it is concluded that the mechanical properties most used in practice are quite sensitive to a change in the state of the WM after its long-term high-temperature degradation in the technological environment of steam piping of TPPs as a hydrogenating environment (Figure 3a). At the same time, the use of the same characteristics to assess the state of the operating BM (steel 15Kh1M1F) looks ineffective (Figure 3b). After all, with the same operation time of the WM and BM on the steam piping, the sensitivity of the analyzed characteristics to the degradation of the WM significantly exceeded their sensitivity to the degradation of the BM (Figure 3). This result was considered as direct evidence of more intensive degradation of WM compared to BM. This conclusion confirms the well-known statement that the WJ is one of the weakest links in welded structures. A more intense degradation of WM relative to BM was associated with the well-known inhomogeneity of the chemical composition of WM and its microstructure, the higher hydrogen content in WM, and the effect of residual stresses inside the WJ [47]. It was also noted that the redistribution of thermal stresses in the pipe section (with active maneuvering of the operating mode of TPP units with a significant number of their shutdowns) could also contribute to the degradation of WM [14]. An important factor intensifying the WM degradation of WM is also its hydrogenation from a high-temperature process environment [33].

### 3.2. Influence of Hydrogenation on the Mechanical Properties of Welded Joint Metal 

#### 3.2.1. Hydrogen Content in the Welded Joint Metal

An important aspect of assessing the serviceability of the WJ of TPP steam piping is to take into account the hydrogen effect on its mechanical properties. The analysis took into account the effect of hydrogen absorbed by the metal during operation on steam piping and additionally charged into the metal during electrolytic hydrogenation to enhance its effect.

It was determined that the integral content of diffusible hydrogen at a temperature of ~900 °C in BM in the initial state was 1 ppm (Table 3). It corresponds to its average content in steel (0.9–1.8 ppm) [48]. WM absorbs hydrogen during welding due to the temperature gradient and the different solubility of hydrogen in liquid and solid states [49]. However, its content in the non-exploited WM was even lower (0.5–0.7 ppm) than in the BM. This is a consequence of the thermal treatment of the WJ, which provides partial desorption of hydrogen from the WM.

An assessment of the hydrogen content in both WJ (in the initial state and after operational degradation) showed that its content in the operated BM increased to 1.3 ppm (i.e., by 30%), and in the WM—up to 1.3–1.8 ppm, which is 2.5–3 times higher than its content in non-exploited WM (Table 3). It is assumed that the macro- and micro-heterogeneity of the WM contributes to the efficient migration of hydrogen. These inhomogeneities contribute to the desorption of hydrogen from the WM during the heat treatment of the WJ, but during operation, they facilitate the hydrogenation of the WM from the process environment. As a result, with the same operating time of the BM and WM on the steam pipeline, the content of hydrogen absorbed by the operated WM significantly exceeded that determined for the operated BM. Moreover, it can be assumed that the hydrogen content in local zones of WM is potentially suitable for the formation of damage along them (grain boundaries or intense slip bands) and could be even higher (due to the high diffusivity of hydrogen) [50]. In this case, the heterogeneity of WM would only contribute to the redistribution of hydrogen inside WJ and, as a consequence, to an increase in its content in zones favorable for the formation of hydrogen traps. As hydrogen accumulates in the microvoids, its pressure will increase, and accordingly, the stresses in WM in their vicinity will also increase [51]. Then, the hydrogen accumulated in the WM can effectively promote its cracking from the inside under the combined action of these additional local stresses and those applied from the outside. Therefore, it was assumed that the higher content of hydrogen absorbed by WM could additionally contribute to its more intense degradation compared to BM. The hydrogen contents in the operated WM and BM are generally consistent with the trend of changes in their mechanical properties, which decrease more strongly in the WM than in the BM (Figure 3).

#### 3.2.2. The Properties of WM after Degassing in Vacuum

**Figure 4:**
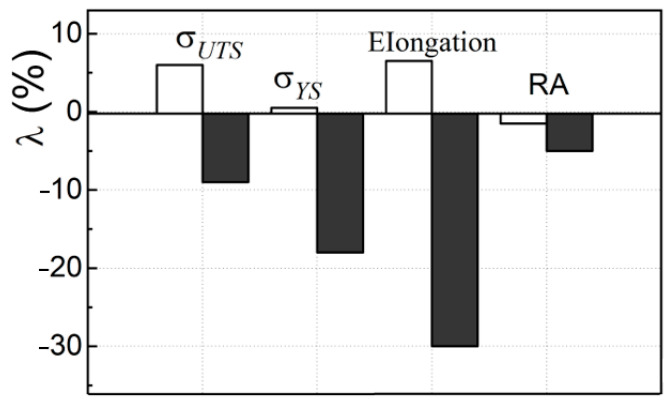
Influence of WM degassing (to remove diffusible internal hydrogen) on its mechanical characteristics in the initial state (white bars) and after ~2 × 10^5^ h of operation on the TPP steam piping (black ones). The coefficient λ characterizes the change in the mechanical properties of WM with internal hydrogen (absorbed during pipe welding or long-term operation) in relation to the corresponding characteristics of the degassed WM (after two hours of holding the specimens in vacuum at a temperature of 570 °C before their tensile test in air).

A significant part of the hydrogen absorbed by the WM during welding and operation on the steam piping or on the hydrogenation reactor could migrate to the zones of the volumetric stress state and, thereby, affect the mechanical properties of the WM during tensile testing of specimens [52,53]. After all, it is known that hydrogen can contribute both to the strengthening of metals (at the macro level) [54] and their plastification (at the micro level) [55]. Such an ambiguous effect of hydrogen is associated with its dual influence on the motion of dislocations [56,57]. To eliminate the effect of hydrogen (available in a non-operating WM or absorbed by it during long-term operation) on the mechanical properties of the WM, some of the specimens were degassed in a vacuum for 2 h at a temperature of 570 °C (that is, the maximum allowable operating temperature of the main steam piping of the TPP) before their tensile tests. As a result, the effect of internal hydrogen absorbed by the WM in both states (before and after operation) on the determined mechanical characteristics was practically excluded (or at least minimized). These characteristics were used to assess the effect of degradation of the exploited WM in its pure form. The WM degradation was evaluated by the coefficient λ characterizing the change in the characteristics of both types of WM (in the initial state and after operation on the steam piping), determined on specimens tested without degassing in vacuum, relative to the corresponding characteristics obtained after their degassing (Figure 4).

The comparison of the properties of WM in the initial state and after operation on steam piping after their additional degassing showed that desorption of hydrogen from WM in the initial state practically does not change its characteristics. At the same time, all properties of an operated WM without it degassing (with hydrogen absorbed by the WM during operation on the steam piping) were lower (σ_UTS_—by 9%, σ_YS_—by 18%, elongation—by 30%, RA—by 6%), than corresponding properties for the WM after desorption of hydrogen from it (Figure 4). Thus, the hydrogen absorbed by the WM had practically no effect on the characteristics of the WM in the initial state but significantly worsened the properties of the operated one. This can be explained by a large number of defects and increased hydrogen content in the operated WM. A synergistic effect of both of these factors is also possible. These data were considered direct evidence of a significant negative effect of internal hydrogen (absorbed by WM during operation) on its defining characteristics.

It was assumed that the degassing of the exploited WM made it possible to assess the change in its mechanical properties only due to degradation. After all, the influence of hydrogen desorption from WM in the initial state had an insignificant effect on its mechanical characteristics, while from the used WM, it was much stronger (Figure 4). Then, the results presented in Figure 3a and Figure 4 allow us to compare the influence of the combined action of degradation and hydrogen in an operating WM and only degradation on its mechanical properties. Obviously, the degradation of the WM in its pure form (without taking into account the influence of internal hydrogen) had an extremely negative impact on all the mechanical characteristics of the operated WM. At the same time, the relative elongation of specimens from operated WM (with hydrogen absorbed by it) increased (with a decrease in all other characteristics). It was assumed that this is the result of the action of hydrogen absorbed by the WM during operation. This hydrogen could promote the stretching of the bridges between the operational defects located along the entire working length of the specimens during their tensile tests. There is also no reason to hope that similar trends in the influence of hydrogen absorbed by WM on its properties will not manifest themselves in the most damaged sections of operating steam piping. Its influence will be especially dangerous during shutdowns of units with cooling of the steam supply system and a decrease in the equilibrium concentration of hydrogen in the metal. In these cases, the hydrogen absorbed by the WM will necessarily contribute to the growth of operational defects. After all, the solubility of hydrogen in a metal decreases with decreasing temperature. This stimulates its diffusion to nearby defects inside the metal as energetically favorable hydrogen traps, which will promote their growth.

#### 3.2.3. Influence of Additional Electrolytic Hydrogenation of Metals from Different WJ Zones on Their Strength Characteristics

**Figure 5:**
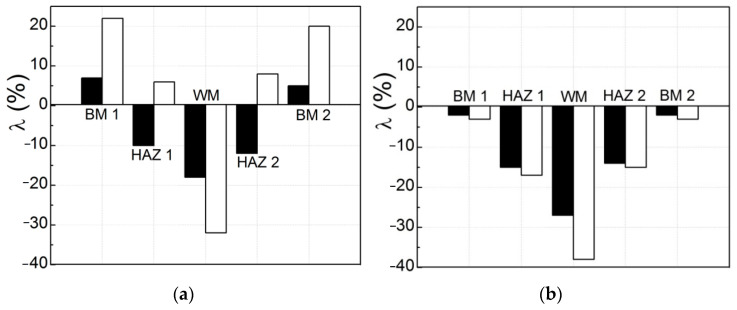
Change in the values of ultimate tensile strength σ_UTS_ (black bars) and yield strength σ_YS_ (white ones) of the metal in the initial state (**a**) and after ~2 × 10^5^ h of operation on the TPP steam piping (**b**). The value of λ characterizes the change in the characteristics of additionally hydrogenated specimens in relation to the corresponding characteristics of specimens without additional hydrogenation.

The possible harmful effect of metal hydrogenation on its strength characteristics is usually not taken into account when calculating the strength of steam piping. However, the study of WM showed the tangibility of its influence on the strength characteristics (especially in exploited WM). Therefore, it was important to evaluate the effect of hydrogenation on the strength characteristics of the metal of different zones (BM, heat-affected zone (HAZ), and WM) of the model weld joint (in the initial state) and after its long-term operation. The specimens were preliminarily hydrogenated by the electrolytic method and then tested for uniaxial tension in air. The effect of internal hydrogen (absorbed by the WM of the model weld during its formation) on its strength did not exceed 6% (Figure 4). Therefore, it was assumed that the effect of internal hydrogen in this series of tests would be mainly related to the hydrogen absorbed by the WJ during additional hydrogenation of the specimens (before their tensile test in air). It has been established that hydrogen, additionally absorbed by the specimens, has an ambiguous effect on the strength of the metal of different zones of the WJ in the initial state (Figure 5a). In particular, the σ_UTS_ values and, to an even greater extent, the σ_YS_ values for BM slightly increased under the influence of hydrogen on both sides of the repair joint (Figure 5a). This is consistent with the possible strengthening effect of hydrogen [54,58]. The effect of hydrogen on the strength characteristics of the HAZ metal and WM of the WJ in the initial state tends to decrease in comparison with the BM. At the same time, the hydrogen absorbed by the metal continued to slightly increase the σ_YS_ values of the BM and HAZ in the WJ in the initial state. Although the values of σ_UTS_ of the HAZ metal on both sides of the WM definitely decreased. At the same time, both strength characteristics (σ_UTS_ and σ_YS_) of WM from WJ in the initial state unambiguously decreased under the influence of hydrogen absorbed by WM during additional hydrogenation. In this case, the negative effect of hydrogen on the ultimate strength σ_UTS_ value was much weaker than on the yield strength σ_YS_ one.

Testing a similar series of specimens from different zones of the operated WJ revealed for all of them an unambiguous decrease in both strength characteristics under the action of an additional portion of hydrogen absorbed by specimens during their electrolytic hydrogenation (Figure 5b). Moreover, this decrease was insignificant for BM (did not exceed 2 … 3%) but was much greater for WM and reached 27% for σ_UTS_ and 38% for σ_YS_. Consequently, the hydrogen absorbed by exploited WJ further reduced all its characteristics under its tensile test in air. This once again confirmed the importance of taking into account the strength loss of WM under the influence of hydrogen absorbed by it (both during welding and operation on TPP steam piping). The influence of hydrogen on the strength characteristics of the WM at the beginning of its operation on steam piping is usually neglected. Our research confirmed the validity of this opinion (at least regarding the OM and HAZ metal). However, its negative impact on the strength characteristics of the metal from all zones of the welded joint (and especially for the weld metal) can become critically dangerous for the safety of steam piping after their long-term operation.

#### 3.2.4. Fractographic Features Induced by Operational Degradation and Hydrogenation of WM

**Figure 6:**
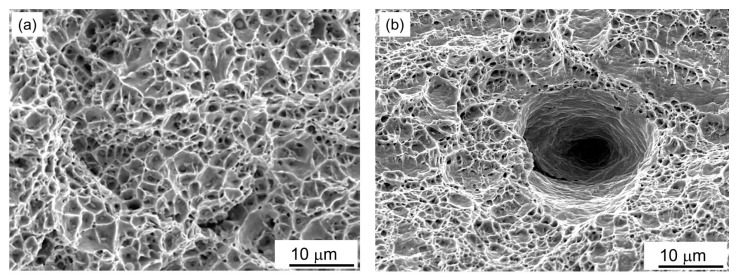
Fractograms of the central part of the fractures of smooth WM specimens in the initial state, tested in the air without preliminary hydrogenation (**a**) and after additional electrolytic hydrogenation before tensile tests in the air (**b**).

**Figure 7:**
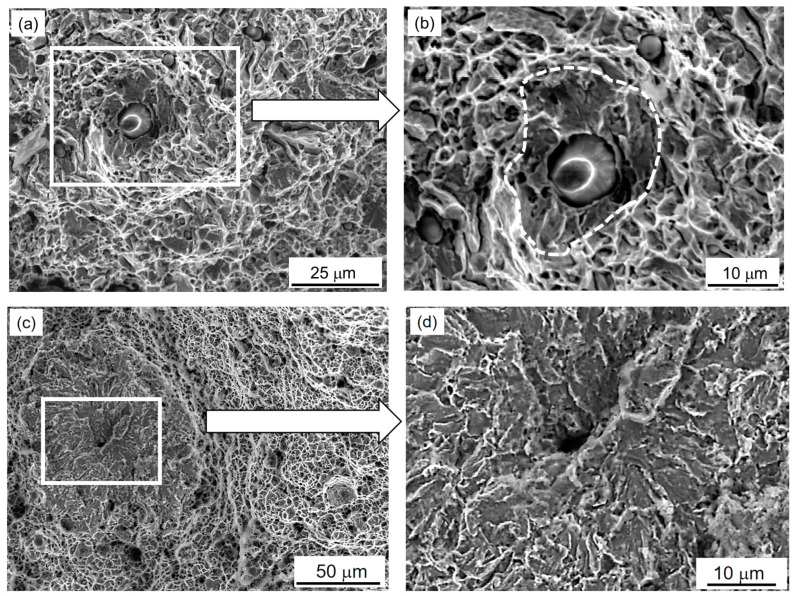
Fractograms obtained at low (**a**,**c**) and high (**b**,**d**) magnifications in the central part of the fractures of smooth WM specimens after ~2 × 10^5^ h operation on TPP steam piping tested in the air without (**a**,**b**) and after their preliminary electrolytic hydrogenation (**c**,**d**). In Figure 7b, the zone of transcrystalline quasi-cleavages is indicated by a dashed line.

Hydrogen absorbed by the metal during electrolytic hydrogenation tends to leave it through the side surfaces of the specimens during their tensile testing in the air. Moreover, the hydrogen can migrate to the nearest structural defects (for example, free surfaces along the interphase boundaries) in their bulk. As a result, microstructure defects (along the boundaries between the matrix phase and various kinds of inclusions, neighboring grains, subgrains, etc.) become favorable traps for hydrogen. Decohesion of inclusions from the matrix promotes the molization of hydrogen in the formed voids and increases its pressure inside them. It was assumed that hydrogen in such traps should leave special fractographic features on the fractures of the specimens, which are not found on the fractures of the non-hydrogenated metal. To identify them, fractographic signs of the fracture surfaces of hydrogenated WM specimens in the initial state and after a long-term operation, tested using tension in the air, were compared (Figure 6 and Figure 7). An exceptionally ductile dimple relief was also observed in the central part of the fractures in the hydrogenated WM specimens in the initial state (Figure 6b). However, against the background of a relief of small dimples at the fracture of the hydrogenated WM, several large and deep voids that formed in place of large non-metallic inclusions were observed. They were considered as a consequence of the decohesion of these large inclusions from the surrounding matrix as a result of the hydrogenation of unexploited WM (Figure 6b). Without hydrogenation of the WM in the initial state, such deep voids practically did not appear during its destruction (Figure 6a). Based on this, it was believed that hydrogen absorbed by WM during electrolytic hydrogenation could contribute to the separation of inclusions from the matrix. However, in general, this feature of hydrogenation of WM in the initial state did not change the ductile dimple mechanism of its destruction.

At the same time, in the central part of the fracture surface (where the fracture is usually initiated during uniaxial tension of smooth samples in air) of a specimen from an operating WM, several local areas of quasi-cleavage were observed (Figure 7a,b). They were visible against the background of the classic relief of ductile dimples around them.

Similar but more clearly defined, rounded areas of quasi-cleavages were also revealed on the fracture of a previously hydrogenated (before testing in air) specimen from an operating WM. Moreover, their number was greater than on the fracture of the non-hydrogenated WM specimen. Small microcleavage areas are usually initiated by a defect at the interface between the matrix and the inclusion (Figure 7b,d). Deep voids were usually located in the center of rounded zones with a predominant brittle cleavage mechanism. Their appearance on the fracture was associated with hydrogen traps, which actually initiated the centrifugal propagation of local brittle cleavages. In the central part of the fractures of the hydrogenated WM specimens in the initial state, such cleavage areas were not observed at all. The tensile tests in the air of both variants of the specimens from the hydrogenated WM (in initial and exploited states) were generally completed with the formation of macroscopically ductile fractures of the cup-cone type with equiaxial dimples in its central parts and typical parabolic ones within their conical parts. However, the cleavage elements found on the fractures of the hydrogenated specimens from the operated WM weakened their working sections, contributed to the localization of deformation in the weakest of them, and contributed to their destruction as a whole.

Based on even a cursory analysis of the traditional dimple relief of the specimens, it was suggested that both the degradation and hydrogenation of WM can contribute to the separation from the matrix of not only large inclusions but also small ones, leaving only their traces at the bottom of the dimples. This was evidenced by some discrepancy between the number of inclusions and their traces at the bottom of the dimples, observed on the fracture surfaces of the WM in different states. If this assumption is true, then the assessment of fractographic features associated with inclusions at the bottom of ductile dimples can be used as a quantitative indicator of the cohesion of inclusions with the matrix. It is also necessary to determine the role of additional WM hydrogenation as a factor contributing to the detachment of inclusions from the matrix [59].

Such an approach would make it possible to quantify the degree of degradation of steels after their long-term operation based on the quantitative analysis of fractographic digital images of fractures of steel specimens in the initial state and after long-term operation [60]. However, its implementation is impossible without the use of computer processing of fractographic digital images. This requires additional research, which is planned to be carried out in the near future.

## 4. Conclusions

It has been demonstrated that, despite the low sensitivity of mechanical characteristics (such as hardness, strength and ductility, and impact strength) to the degradation of the base metal, all these characteristics showed a rather high sensitivity to the operational degradation of the WM. In the initial state, the characteristics of WM exceeded the corresponding indicators of base metal. However, already, after ~2 × 10^5^ h of operation, the hardness and impact strength of WM became even lower than the allowable values for BM. After the operation of WM on the steam piping, its impact strength KCV decreased by 86%, tensile strength σ_UTS_ by 26%, and yield strength σ_YS_ by 44%. At the same time, the plasticity characteristics of WM changed ambiguously (RA decreased by 16%, and elongation increased by 33%). An unusual opposite change in both ductility indicators and a simultaneous decrease in hardness and strength, characterizing the resistance to ductile fracture, and impact strength, characterizing the resistance to brittle fracture, were considered a consequence and a distinctive feature of the operational degradation of WM.

It has been established that after the operation of the weld on the steam piping, the integral content of hydrogen in the BM increased by 30% and in the WM—by three times. Such a high content of hydrogen in the operated WM was considered a factor contributing to its more intensive degradation. Vacuum degassing of WM in the initial state showed an insignificant effect of hydrogen absorbed during welding on its strength and ductility characteristics. However, after the degassing of the operating WM (when the role of the hydrogen absorbed by it is reduced to a minimum), a clear degradation’s negative effect on its mechanical properties during tensile tests was shown.

It is shown that additional electrolytic hydrogenation of specimens of all zones of the WJ before tensile testing in the air will increase the sensitivity of all mechanical characteristics to metal degradation. As a result of hydrogenation, the mechanical properties of the WM are most significantly reduced, which indicates an increased susceptibility of exploited WM to hydrogen embrittlement. These results substantiated the possibility of using the relative change in the strength characteristics of additionally hydrogenated WM to assess the degree of its operational degradation.

In the central part of the fracture surface of the operational WM specimen, which was additionally hydrogenated before the tensile test in air, rounded areas of local transcrystalline cleavages were found. They were clearly visible against the background of classical ductile dimple fractures. Deep voids in the center of these cleavage areas were considered as fractographically visualized hydrogen traps at defects along the matrix boundary with inclusions. Similar areas of brittle cleavage were not detected at the fractures of the hydrogenated WM specimen in the initial state. Therefore, it was assumed that the additional hydrogenation of the previously operated WM contributed to the fractographic visualization of operational defects in it.

## Figures and Tables

**Table 1:** The chemical composition of the WM of both studied welded joints (wt.%).

	C	Si	Mn	S	P	Cr	Mo	V	Cu	Ni	Al	Co
Initial state	0.09	0.18	0.69	0.009	0.021	1.13	0.57	0.19	0.1	0.07	0.01	0.01
Exploited state	0.05	0.34	1.09	0.016	0.031	1.06	0.68	0.2	0.21	0.18	0.01	0.02

**Table 2:** Mechanical properties of the metal WJ in the initial state.

	Steel	σ_UTS_ (MPa)	σ_YS_ (MPa)	RA (%)	Elongation (%)	HB
BM	15Kh1M1F	530	340	63	20.1	167
WM	09Kh1MF	622	450	77	18.7	242

**Table 3:** Hydrogen content in the WJ of pipes made of steel 15Kh1M1F in the initial state and after ~2 × 10^5^ h of operation on the TPP main steam piping.

Metal State	Hydrogen Content (ppm)	
	WM	BM
Initial	0.45–0.72	0.99
Exploited	1.26–1.80	1.26

## Data Availability

Data are contained within the article.
